# BODIPY-Based Fluorescent Probes for Selective Visualization of Endogenous Hypochlorous Acid in Living Cells via Triazolopyridine Formation

**DOI:** 10.3390/bios12110923

**Published:** 2022-10-25

**Authors:** Peraya Hiranmartsuwan, Sirilak Wangngae, Jukkrit Nootem, Anyanee Kamkaew, Rathawat Daengngern, Worawat Wattanathana, Kantapat Chansaenpak

**Affiliations:** 1 National Nanotechnology Center, National Science and Technology Development Agency, Thailand Science Park, Pathum Thani 12120, Thailand; 2 School of Chemistry, Institute of Science, Suranaree University of Technology, Nakhon Ratchasima 30000, Thailand; 3 Integrated Applied Chemistry Research Unit, King Mongkut’s Institute of Technology Ladkrabang, School of Science, Bangkok 10520, Thailand; 4 Department of Materials Engineering, Faculty of Engineering, Kasetsart University, Ladyao, Chatuchak, Bangkok 10900, Thailand

**Keywords:** fluorescence, chemosensor, hypochlorous Acid, BODIPY, bioimaging

## Abstract

In this work, the two pyridylhydrazone-tethered BODIPY compounds (**2** and **3**) were synthesized. These compounds aimed to detect hypochlorous acid (HOCl) species via cyclic triazolopyridine formation. The open forms and the resulting cyclic forms of BODIPYs (**2**, **3**, **4**, and **5**) were fully characterized by nuclear magnetic resonance, mass spectrometry, infrared spectroscopy, and single-crystal X-ray diffraction. These two probes can selectively detect HOCl through a fluorescence turn-on mechanism with the limit of detections of 0.21 µM and 0.77 µM for compounds **2** and **3**, respectively. This fluorescence enhancement phenomenon could be the effect from C = N isomerization inhibition due to HOCl-triggered triazolopyridine formation. In cell imaging experiments, these compounds showed excellent biocompatibility toward RAW 264.7 murine live macrophage cells and greatly visualized endogenous HOCl in living cells stimulated with lipopolysaccharide.

## 1. Introduction

Hypochlorous acid (HOCl) is an oxidant widely used as a composition in disinfectant for water supplies, household bleach, and antimicrobial agents. It can partially dissociate to hypochlorite (OCl^-)^ species in aqueous solution at physiological condition. In addition, it can be used as disinfectant against COVID-19 (Coronavirus Disease 2019) virus on non-porous surfaces approved by the Environmental Protection Association (EPA) [1,2]. It is also one of the essential reactive oxygen species (ROS) in diverse physiological and pathological processes [3,4]. Typically, endogenous HOCl is generated from hydrogen peroxide and chloride anions, catalyzed by myeloperoxidase in neutrophils, a type of white blood cells, as a part of the innate host defense system for invading pathogens and microbes [5,6,7]. Nevertheless, the excessive HOCl in vivo can cause several diseases linked to oxidative stress injury, such as aging [8], inflammation [9], neurodegenerative disorders [10], and cancer [6]. Therefore, the efficient tool for detection and monitoring of HOCl in cellular level as well as other complex biosystems is of special interest for biological research and clinical diagnosis.

**Figure 1:**
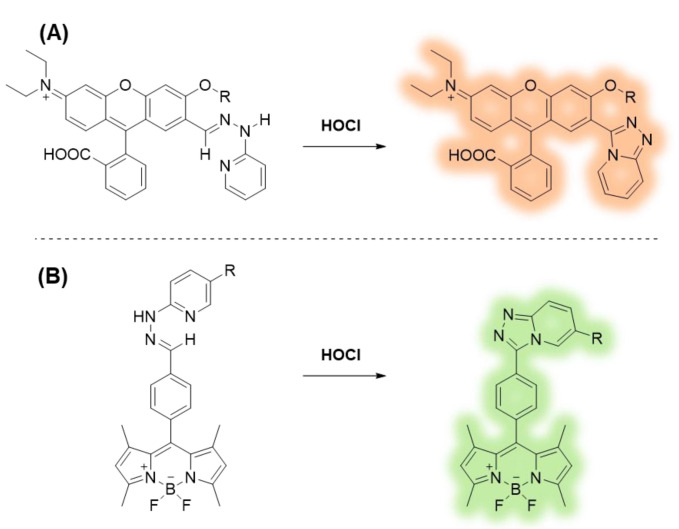
HOCl-triggered triazolopyridine formation reactions of the rhodol backbone in the literature (**A**) and the BODIPY backbone in this work (**B**).

Various methods have been reported for HOCl sensing, such as electrochemical techniques [11,12], spectrophotometry-based flow injection analysis [13], and test strips based on chemiluminescence [14]. These methods are simple, cost-effective, highly selective, and sensitive toward HOCl/OCl^-^ species. However, those techniques are not applicable for HOCl sensing in biological samples. Currently, fluorescence methods for HOCl detection using designed molecular probes have attracted high interest due to favorable spatiotemporal resolution in living systems. HOCl sensors have been developed in many fluorophores, such as naphthalimides [15,16,17], rhodamines [18,19,20], fluoresceins [21,22,23], coumarins [24,25,26], and BODIPYs [27,28,29]. As seen in the literature, most emissive HOCl sensors rely on a reaction-based strategy due to the powerful oxidizing nature of HOCl [30,31,32]. Those reactions include deformylation [33], deoximation [34,35], oxidation of thioethers [36,37,38], oxidation of selenides [39,40,41], and chlorination of thioesters [42,43,44]. Recently, Guo’s research team has invented a new reaction based on HOCl-triggered triazolopyridine formation [45,46]. This reaction has been systematically employed on the rhodol backbone (Figure 1A) for selective detection of HOCl and even created as a mitochondria-specific fluorescent probe that could visualize HOCl in living cells and zebrafish. In this work, we expanded this HOCl-sensing strategy to the BODIPY derivatives (Figure 1B) and confirmed their potentials in the imaging of endogenous HOCl in cells. As BODIPY probes can be simply modified as cancer photo-therapeutic agents [47], the introduction of HOCl-detection function to BODIPY backbone could lead to a new direction in the development of new dual-mode probes for cancer imaging and therapy. The detailed synthesis steps and photophysical properties of these compounds are presented in this publication.

## 2. Materials and Methods

### 2.1. Materials and Instruments

Compounds **1** and **2** were synthesized according to the reports in the literature [48,49,50]. 2-Hydrazinopyridine, 2-hydrazino-5-iodopyridine, Dess–Martin periodinane, 2,3-dichloro-5,6-dicyanoquinone (DDQ), 2,4-dimethylpyrrole, sodium hypochlorite, and glacial acetic acid were purchased from Tokyo Chemical Industry (TCI), Tokyo, Japan; boron trifluoride diethyl etherate purified by redistillation, and 4-(hydroxymethyl)benzaldehyde dimethyl acetal were purchased from Sigma-Aldrich, St. Louis, MO, USA; organic solvents, including dichloromethane (DCM), tetrahydrofuran (THF), and ethanol (ACS grade) were purchased from Honeywell, Charlotte, NC, USA. 12). All chemicals were used without additional purifications. Electrospray mass spectra were obtained from a Bruker micrOTOF spectrometer (Bruker, Billerica, MA, USA). At room temperature, NMR spectra were recorded on a Bruker Avance 500 MHz NMR spectrometer (Bruker, Billerica, MA, USA). The solvent chemical shifts were recorded and reported in ppm (DMSO-d_6_ at 2.50 ppm for ^1^H NMR and 39.51 ppm for ^13^C NMR). Data were reported as follows: chemical shift, multiplicity (s = singlet, d = doublet, t = triplet), coupling constant (J), and the number of protons. Fourier transform infrared spectroscopy (FTIR) spectra were acquired from an IRTracer-100 spectrophotometer (SHIMADZU, Kyoto, Japan). The X-ray crystallographic measurement was performed using a Bruker D8 Venture diffractometer (Bruker, Billerica, MA, USA). UV-VIS absorption and fluorescence spectra were acquired from a Cary Series UV-Vis-NIR spectrophotometer (Agilent Tech, Santa Clara, CA, USA) and a Perkin Elmer LS55 fluorescence spectrometer (PerkinElmer, Waltham, MA, USA), respectively.

### 2.2. Synthetic Procedures

Synthesis of (E)-5,5-difluoro-10-(4-((2-(5-iodopyridin-2-yl)hydrazono)methyl)phenyl)-1,3,7,9-tetramethyl-5H-4λ^4^,5λ^4^-dipyrrolo [1,2-c:2’,1’-f][1,3,2]diazaborinine (**3**).

A solution of compound **1** (0.57 mmol) and 2-hydrazino-5-iodopyridine (1.14 mmol) was refluxed in ethanol (10 mL) at 90 °C for 5 h in the presence of a catalytic amount of acetic acid. After solvent evaporation, the obtained solid was washed with cool ethanol twice. The solid was further purified by column chromatography with DCM as an eluent to get an orange solid in an 86% yield. ^1^H NMR (500 MHz, DMSO-d_6_) δ 11.17 (s, 1H), 8.30 (s, 1H), 8.10 (s, 1H), 7.91 (d, J = 7.1 Hz, 1H), 7.85 (d, J = 7.5 Hz, 2H), 7.40 (d, J = 7.5 Hz, 2H), 7.19 (d, J = 8.6 Hz, 1H), 6.19 (s, 2H). 2.45 (s, 6H), 1.41 (s, 6H). ^13^C NMR (125 MHz, DMSO-d_6_) δ 155.96, 154.95, 153.05, 145.40, 142.67, 141.56, 138.81, 135.96, 134.06, 130.59, 128.31, 126.77, 121.42, 109.11, 79.95, 14.21, 14.14. HRMS (ESI), m/z calcd for C_25_H_24_BF_2_IN_5_ ([M+H]^+^): 570.1137, found: 570.1132.

Synthesis of 10-(4-([1,2,4]triazolo[4,3-a]pyridin-3-yl)phenyl)-5,5-difluoro-1,3,7,9-tetramethyl-5H-4λ^4^,5λ^4^-dipyrrolo[1,2-c:2’,1’-f][1,3,2]diazaborinine (**4**).

Compound **2** (0.49 mmol) was dissolved in 10 mL of THF. After that, 2 mL of 0.5 M sodium hypochlorite solution was added into the solution, followed by stirring at room temperature for 4 h. Then, THF was evaporated under reduced pressure and the crude was extracted with DCM (2 × 10 mL). The organic layer was washed twice with DI water (2 × 10 mL) and the product was purified by column chromatography with haxance/EtOAc (1:0 to 0:1) as an eluent to obtain a brown solid in a 37.2% yield. ^1^H NMR (500 MHz, DMSO-d_6_) δ 8.62 (d, J = 6.8 Hz, 1H), 8.08 (d, J = 7.8 Hz, 2H), 7.86 (d, J = 9.2 Hz, 1H), 7.61 (d, J = 7.8 Hz, 2H), 7.44 (t, J = 6.8 Hz, 1H), 7.05 (t, J = 6.8 Hz, 1H), 6.19 (s, 2H), 2.44 (s, 6H), 1.43 (s, 6H). ^13^C NMR (125 MHz, DMSO-d_6_) δ 155.18, 150.19, 145.38, 142.76, 140.95, 135.42, 130.55, 128.88, 128.76, 128.11, 127.34, 123.98, 121.57, 115.72, 114.73, 14.24. HRMS (ESI), m/z calcd for C_25_H_23_BF_2_N_5_ ([M+H]^+^): 442.2014, found: 442.2017.

Synthesis of 5,5-difluoro-10-(4-(6-iodo-[1,2,4]triazolo[4,3-a]pyridin-3-yl)phenyl)-1,3,7,9-tetramethyl-5H-4λ^4^,5λ^4^-dipyrrolo[1,2-c:2’,1’-f][1,3,2]diazaborinine (**5**).

Compound **3** (0.18 mmol) was dissolved in 5 mL of THF. After that, 1.5 mL of 0.5 M sodium hypochlorite solution was added into the solution, followed by stirring at room temperature for 4 h. Then, THF was evaporated under reduced pressure and the crude was extracted with DCM (2 x 10 mL). The organic layer was washed twice with DI water (2 x 10 mL) and the obtained product was purified by column chromatography with 1:4 EtOAc/DCM as an eluent to get a brown solid in a 61.1% yield. ^1^H NMR (500 MHz, DMSO-d_6_) δ 8.83 (s, 1H), 8.13 (d, J = 7.7 Hz, 2H), 7.75 (d, J = 9.3 Hz, 1H), 7.64 (d, J = 7.9 Hz, 3H), 6.23 (s, 2H), 2.48 (s, 6H), 1.46 (s, 6H). ^13^C NMR (125 MHz, DMSO-d_6_) δ 155.17, 148.96, 145.02, 142.77, 140.97, 135.71, 135.54, 130.53, 128.88, 128.12, 126.95, 121.54, 116.83, 79.94, 14.32, 14.25. HRMS (ESI), m/z calcd for C_25_H_22_BF_2_IN_5_ ([M+H]^+^): 568.0976, found: 568.0985.

### 2.3. Crystallographic Measurements

Single crystals of compound **4** was obtained by slow evaporation of pentane into its solution in tetrahydrofuran solvent. The crystallographic measurement was performed using a Bruker D8 Venture diffractometer with graphite-monochromated Cu-Kα radiation. The semi-empirical method *SADABS* was performed for an absorption correction [51] and the data reduction was carried out by *SAINT* [52]. Using Olex2 software [53], the crystal structure was solved by the intrinsic phasing method using *SHELXT* [54] and refined by *SHELXL* [55], employing a full-matrix least-squares method against *F^2^* with the anisotropic temperature parameters for all non-hydrogen atoms. All atoms were geometrically placed and refined using the riding model approximation. Mercury software [56] was used to provide graphics regarding crystal structure and intermolecular interaction. The crystallographic data of compound **4** were deposited in the Cambridge Crystallographic Data Center, deposition number (CCDC No.) 2211127.

### 2.4. Fluorescence Quantum Yields Calculations

Fluorescence quantum yields of the compounds **2**, **3**, **4**, and **5** were measured in MeOH-5mM Phosphate buffer solution (PBS) (1:1 *v*/*v*) using fluorescein in 0.1M NaOH solution as a standard (*Q*_*std*_ = 0.95) and were calculated based on the Equation (**1**):

(1) $$Q=Q_{std}\times \left(\frac{I_{sample}}{I_{std}}\right)\times \left(\frac{A_{std}}{A_{sample}}\right)\times {\left(\frac{\eta_{sample}}{\eta_{std}}\right)}^{2}$$

where, *Q* denotes the fluorescence quantum yields, *I* is the peak area of emission spectra, *A* is absorption intensities at the excitation wavelength, and *ŋ* is the solvent reflective index.

### 2.5. Theoretical Calculations

To understand the electronic and photophysical properties of the BODIPY compounds, a Density Functional Theory (DFT) at M062X [57] was performed in an implicit aqueous solution using a conductor-like polarized continuum model (C-PCM) framework [58,59]. The basis set of 6-311G* was applied for all atoms except the iodine (I) atom. In the case of I atom, LANL2DZ effective core potential was applied to describe the I core electrons. The frontier molecular orbitals (FMOs) and HOMO–LUMO energy diagrams were also calculated. All calculations were performed by the Gaussian 16 program package [60].

### 2.6. Cell Viability Assay

RAW264.7 cells were seeded into 96-well cell culture plates at 1 × 10^4^/well and incubated for 24 h before being treated with compounds **2** and **3** at various concentrations (0, 2.5, 5, 10, 20, 30, 50, and 100 µM). The cell viability was measured after 24 h exposure to the compounds using the standard MTT protocol. In short, the cells were treated with methylthiazolyldiphenyl-tetrazolium bromide (200 µL, 0.5 mg/mL, Sigma-Aldrich) for 2 h after being rinsed with PBS three times. Then, culture media were substituted with DMSO, and cell viability was assessed using a microplate reader (BMG Labtech/SPECTROstar Nano).

### 2.7. Cell Culture and Imaging Experiment

*Cell culture*: Dulbecco’s Modified Eagle Medium/High Glucose (DMEM/HIGH GLUCOSE, GE Healthcare Life Sciences HyClone Laboratories), supplemented with 10% fetal bovine serum (FBS, Gibco), and 1% Penicillin-Streptomycin Solution, was used to cultivate murine macrophage cells (RAW264.7, ATCC) on 75 cm^2^ culture flasks (CORNING) at 37 °C and 5% CO_2_ humidified incubator.

*Cell imaging*: RAW264.7 cells were seeded at a density of 7 × 10^3^ cells per well in an 8-well Chambered Coverglass with non-removable wells (Nunc Lab-Tek II Chamber Slide) and incubated for 24 h. For the endogenous HOCl imaging, the RAW 264.7 cells were stimulated by lipopolysaccharides (LPS). In brief, the RAW cells were incubated with 1 μM of compounds **2** and **3** in DMEM with 5 % FBS for 30 min followed by incubation with LPS (5 µg/mL) for 2 h. Then, the cells were washed three times with PBS buffer. For the exogenous HOCl imaging, the cells were treated with 1 μM of compounds **2** and **3** for 30 min followed by incubation with NaOCl (20 μM) for another 30 min. After that, the cells were washed three times with PBS. All the cells were brought to image under Laser Scanning Confocal Microscope (LSCM, Nikon A1Rsi). Compounds **2** and **3** were excited by a 488 nm laser. A 60 X oil immersion objective lens was used in these experiments

## 3. Results and Discussion

### 3.1. Synthesis and HOCl Response of BODIPY Probes

**Scheme 1:**
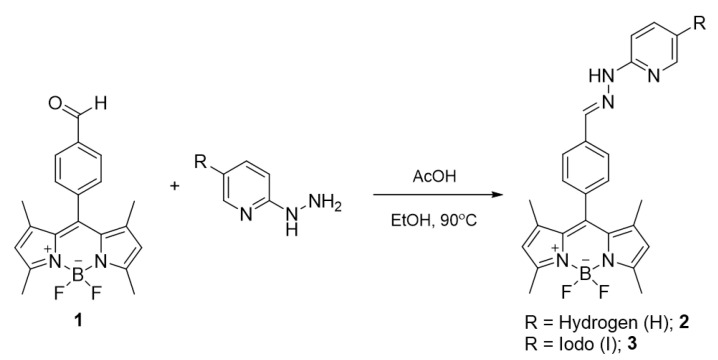
The synthesis of BODIPY probes.

The BODIPY probes were synthesized by acid-catalyzed condensation reactions between the aldehyde-linked BODIPY (**1**) and 2-hydrazinopyridine/2-hydrazino-5-iodopyridine yielding compounds **2** and **3**, respectively (Scheme 1). The BODIPYs were fully characterized by nuclear magnetic resonance spectroscopy (^1^H NMR and ^13^C NMR), and mass spectrometry (MS) (Figures S1,S2,S7, Supplementary Materials) and the resulting spectra matched well with the results in the previous literature [50].

**Scheme 2:**
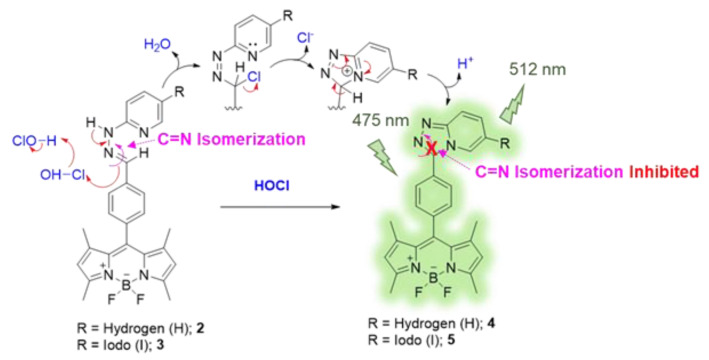
The proposed HOCl-sensing mechanism of the BODIPY probes.

The HOCl-responsive property of these BODIPY derivatives was then investigated. Typically, compounds **2** and **3** displayed a poor fluorescence signal due to C = N isomerization. The C = N isomerization and rotation could lead to an energy decay phenomenon in the excited state of the BODIPY compounds resulting in energy loss, and then quenched the fluorescence emission of the probes [61]. Upon addition of HOCl, the pyridylhydrazone unit of **2** and **3** could be converted to 1,2,4-triazolo[4,3-a]pyridine moiety by the chlorination-induced cyclization [45,46]. The possible reaction mechanism is displayed in Scheme 2. The Cl^+^ from HOCl could undergo electrophilic addition at the C = N bond of the hydrazone unit. The pyridine nitrogen then attacked the chloro-substituted carbon atom yielding cationic triazolopyridinium cyclic intermediate. The following deprotonation led to the formation of triazolopyridine structure (1,2,4-triazolo[4,3-a]pyridine) causing fluorescence enhancement as the C = N isomerization was inhibited. The HOCl-mediated cyclization products (compounds **4** and **5**) were isolated and systematically characterized by NMR and MS techniques as shown in Figures S3–S6, S8 and S9.

**Figure 2:**
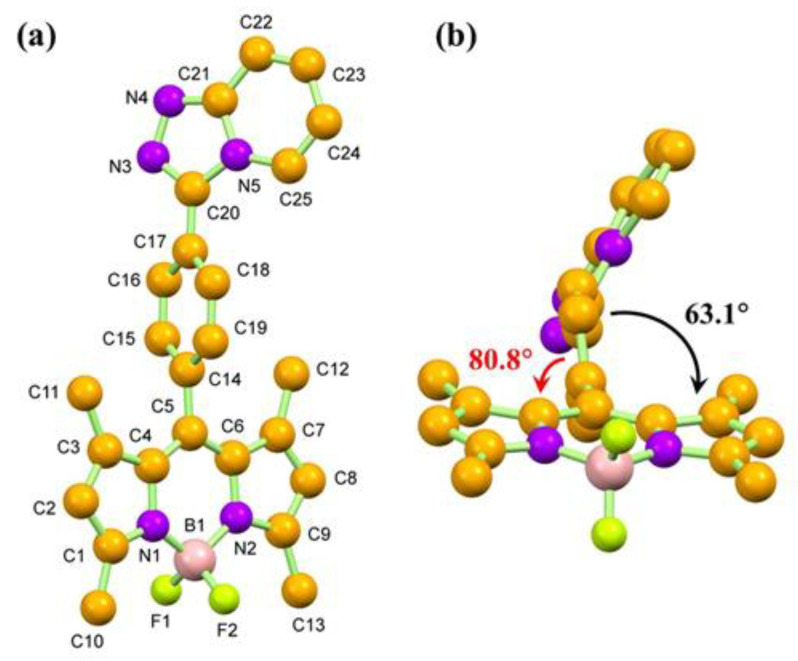
Molecular structures of compound **4** in top-view (**a**) and side-view (**b**). Non-IUPAC atomic numbering is used, and all the hydrogen atoms are omitted for simplification.

The structure of compound **4** was further confirmed by single crystal X-ray crystallographic technique. Compound **4** crystallized in the triclinic system containing two molecules per unit cell. The molecular structure of compound **4** is illustrated in Figure 2a, while crystal data and the corresponding refinement detail are tabulated in Table S1. Within the compound, the BODIPY (C1-C9/N1-N2/B1) moiety, comprised of a central six-membered and two neighboring five-membered rings, is essentially flat. The minimum and maximum deviations from the least-squares BODIPY mean plane is 0.000(2) Å for the N2 atom and -0.073(3) Å for B1 atom, respectively. All the C−C and C−N bonds of the BODIPY group are similar to those reports in the literature [62]. Interestingly, the C5−C14 bond (1.495(4) Å) is longer than those of other C(sp^2^) −C(sp^2^) bonds. This suggests that the delocalized π-system is disturbed at the position. As observed from the side view of the molecule of compound **4** (Figure 1b), the phenyl triazolopyridine group is twisted from the BODIPY plane to reduce the steric effect since the torsion angle between the BODIPY and the phenyl spacer (C14–C19) mean planes is 80.8°. This might be the reason for the disturbance of the π system, resulting in the extension of the C5−C14 bond. Moreover, the angle between the BODIPY and the triazolopyridine is 63.1°, which is in agreement with the values of the compounds with similar structures [62].

**Figure 3:**
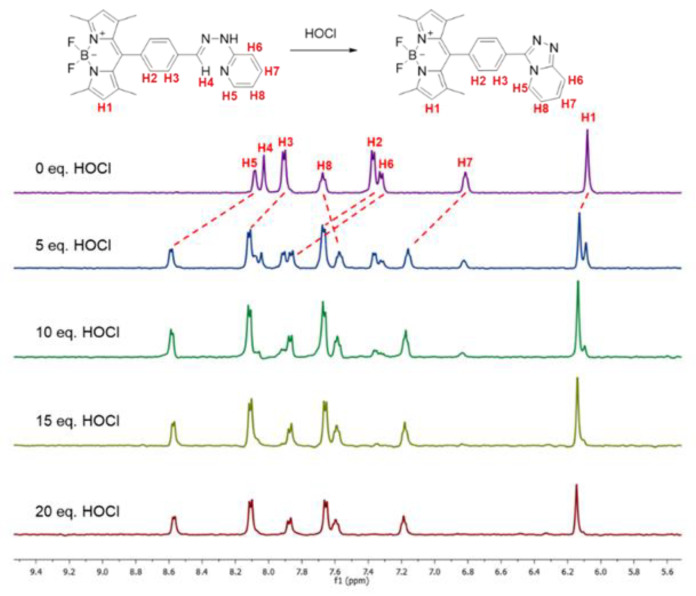
^1^H NMR (500 MHz) spectra of compound **2** in the presence of HOCl in CD_3_OD.

Next, the formation of triazolopyridine unit of the BODIPY probe was investigated by ^1^H NMR and Fourier transform infrared (FTIR) spectroscopy using compound **2** as a model. In the ^1^H NMR titration experiment demonstrated in Figure 3, the hydrazone proton resonance (H_4_) at 8.03 ppm was gradually disappeared upon addition of HOCl, indicating a deprotonation process. In addition, most of the proton resonances in the aromatic region, including pyridyl protons (H_5_, H_6_, and H_7_) and phenyl protons (H_2_ and H_3_), were substantially shifted downfield due to an electron withdrawing effect from the five-membered 1,2,4-triazole ring through π-conjugation. The proton resonance on the BODIPY ring (H_1_) was slightly moved downfield due to a minimal electronic effect from the triazolopyridine moiety. The NMR pattern of the HOCl-titrating product is consistent with that of compound **4,** confirming the abovementioned mechanism.

Fourier transform infrared spectroscopy (FTIR) spectra of compound **2** before and after HOCl treatment are displayed in Figure S12. According to the FTIR result, the N-H stretching vibration is found at 3292 cm^−1^ in **2** without HOCl treatment. After HOCl addition, the N-H vibration is removed. Moreover, the peaks at 1730 and 1631 cm^−1^ are also recognized in the spectrum of compound **4** due to the newly formed C=N bonds of the triazolopyridine group. This supports the formation of the cyclic structure for compound **4**. The results are in line with the NMR and SC-XRD techniques.

### 3.2. Photophysical and HOCl-Sensing Properties of the BODIPY Probes

In order to find the suitable solvent system for HOCl detection, the HOCl-responsive property of compound **2** was first investigated in water-miscible solvents, such as acetonitrile (ACN), ethanol (EtOH), and methanol (MeOH). As shown in Figure S10, compound **2** demonstrated distinct fluorescence enhancement upon addition of HOCl in MeOH, while displayed negligible changes in fluorescent intensities in ACN and EtOH. Therefore, the MeOH solvent is selected as a co-solvent for aqueous media used for HOCl sensing. As seen in Figure S11, compound **2** displayed drastic fluorescence amplification upon adding HOCl in 50% DI water-MeOH mixture. Based on this data, the further HOCl-sensing experiments of the BODIPY probes would be carried out in MeOH-5mM PBS (1:1 *v*/*v*) for the sake of acquiring suitable fluorescent sensitivity and pH control. Gratifyingly, both compounds **2** and **3** exhibited fast response toward HOCl as their fluorescent signals reached saturation within 2 min in this solvent system (Figure S14).

**Figure 4:**
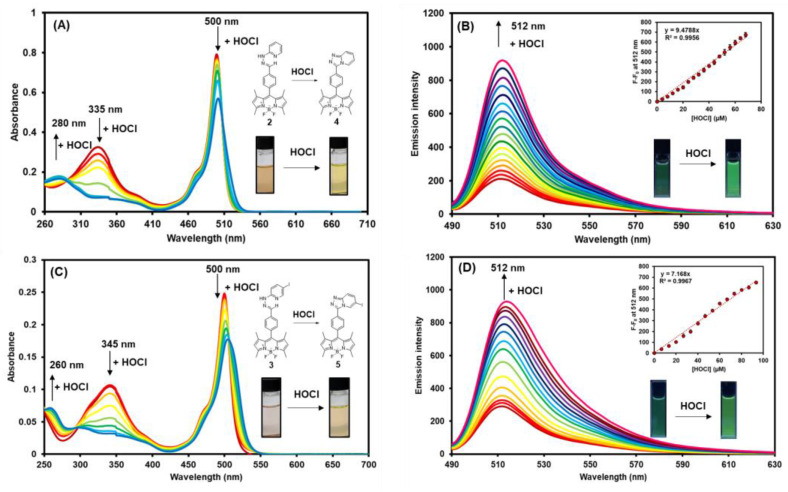
The changes in the absorption spectra of compound **2** (**A**) and compound **3** (**C**), and the changes in emission spectra of compound **2** (**B**) and compound **3** (**D**) (excitation wavelength = 475 nm) in the presence of various concentrations of HOCl in MeOH-5mM PBS (1:1 *v/v*) (final concentrations of BODIPY compounds = 0.2 µM). Error bars represent standard deviation (*n* = 3).

Next, the HOCl titrations with compounds **2** and **3** were investigated by absorption and fluorescence spectroscopy. As seen in Figure 4A,C, upon adding HOCl, the absorption intensities of compound **2** at 500 nm and 335 nm gradually decreased while the one at 280 nm constantly increased. Likewise, the absorption intensities of compound **3** at 500 nm and 345 nm decreased while the intensity at 260 nm increased. It is worth noting that the absorption maximum at 500 nm of compound **3** was slightly redshifted to 506 nm upon addition of HOCl due to the heavy atom effect from an iodine atom [63]. In the fluorescent titration studies, both BODIPY compounds showed a considerable fluorescence enhancement at 512 nm in the presence of HOCl with the small redshift in the case of compound **3** (Figure 4B,D). These results are consistent with the calculated fluorescence quantum yield (QY) values demonstrated in Table 1. The QYs of the HOCl-mediated cyclization products, compounds **4** and **5**, are about 10 times and 5 times higher than those of compounds **2** and **3**, respectively. These results suggest a great potential of these BODIPY derivatives as fluorescence “turn-on” sensors for hypochlorous acid in aqueous media and biological matrices.

**Figure 5:**
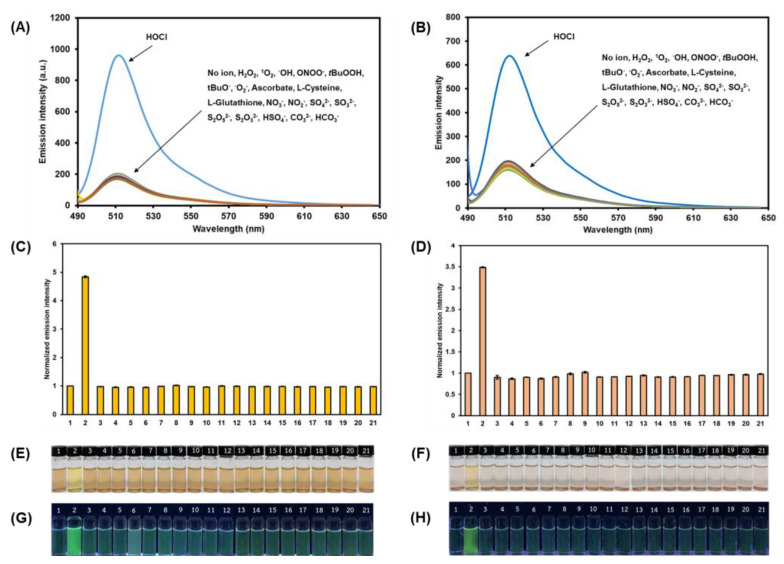
Emission spectra of compound **2** (**A**) and compound **3** (**B**) in the presence of various analytes in MeOH—5 mM PBS (1:1 *v/v*). Bar charts demonstrating fluorescence intensities at 512 nm of compound **2** (**C**) and compound **3** (**D**) in the presence of various analytes. Comparison of the color of BODIPY solutions (compound **2** (**E**) and compound **3** (**F**)) and the visual emission under UV light (compound **2** (G) and compound **3** (**H**)) in the presence of various analytes. [BODIPYs] = 0.2 µM, [Analytes] = 40 µM. 1: blank, 2: HOCl, 3: H_2_O_2_, 4: ^1^O_2_, 5: ^·^OH, 6: ONOO^-^, 7: *t*BuOOH, 8: *t*BuO^·^, 9: ^·^O_2_^−^, 10: Ascorbate, 11: L-Cysteine, 12: L-Glutathione, 13: NO_3_^-^, 14: NO_2_^-^, 15: SO_4_^−^, 16: SO_3_^2−^, 17: S_2_O_5_^2−^, 18: S_2_O_3_^2−^, 19: HSO_4_^−^, 20: CO_3_^2−^, 21: HCO_3_^−^. Error bars represent standard deviation (*n* = 3).

As displayed in the insets of Figure 5B,D, the emission intensities of compound **2** and **3** showed a satisfactory linear relationship with the concentrations of HOCl added. Based on these linear regression curves, the limit of detection (LOD) of probes **2** and **3** were calculated as 0.21 µM and 0.77 µM, respectively, by applying 3σ/*m* formula, where σ is the standard deviation (SD) of the fluorescence signal of blank samples while *m* is the slopes of the linear curves. Gratifyingly, the LODs of compounds **2** and **3** were comparable to or lower than the other BODIPY-based HOCl/ClO^-^-sensing probes reported in the literature (Table 2). In terms of molecular design, compounds **2** and **3** with a pyridylhydrazone unit can effectively detect HOCl via triazolopyridine formation, which is an “eco-friendly” reaction. They did not release additional substances, except H_2_O, upon cyclization, while the other BODIPY probes (Table 2) that detected HOCl based on the deoximation, the desulfurization, and the removal of 2,4-dinitrophenyl hydrazine, etc., produced additional by-products to the sensing systems. It is also worth mentioning that the hypochlorous/hypochlorite detections of most reported HOCl/ClO^-^-responsive probes were carried out in aqueous solution with 50% organic water-miscible co-solvents, such as methanol, ethanol, tetrahydrofuran, acetonitrile, dimethylformamide, etc. [32,64]. Additionally, our developed BODIPY-based compounds (compounds **2** and **3**) exhibited better fluorescence quantum yields (Φ_f_) than the model compounds, the rhodol-based probes that detected HOCl through a triazolopyridine formation, after adding HOCl (Table S2). However, those rhodol derivatives showed lower limits of detection (LOD) than our BODIPY-based sensors. Therefore, the future research will focus on lowering the LODs of the BODIPY probes, which could be done by the introduction of the hydrophilic moiety to the molecules.

Subsequently, the selectivity of BODIPY probes **2** and **3** toward HOCl was assessed in MeOH—5 mM PBS (1:1 *v/v*). As displayed in Figure 5A–D, the fluorescence emissions of compounds **2** and **3** were significantly enhanced upon the addition of HOCl. On the other hand, the fluorescent signals of **2** and **3** manifested negligible changes after the addition of other potential competing species, including, hydrogen peroxide (H_2_O_2_), singlet oxygen (^1^O_2_), hydroxyl radical (^·^OH), peroxynitrite ion (ONOO^-^), *tert*-butyl hydroperoxide (*t*BuOOH), *tert*-butoxyl radical (*t*BuO^·^), superoxide ion (^·^O_2_^−^), ascorbate, L-cysteine, L-glutathione, nitrate ion (NO_3_^-^), nitrite ion (NO_2_^−^), sulfate ion (SO_4_^2−^), sulfite ion (SO_3_^2−^), disulfite ion (S_2_O_5_^2−^), thiosulfate ion (S_2_O_3_^2−^), hydrogen sulfate ion (HSO_4_^−^), carbonate ion (CO_3_^2−^), and hydrogen carbonate ion (HCO_3_^−^). In addition, the selective visualization of HOCl of compounds **2** and **3** over other competitive species was indicated by the change of the solution color and fluorescent signal, which could be noticed by naked eyes (Figure 5E–H). Upon adding HOCl, the colors of the solutions were changed from yellow to brownish orange for **2** and pale pink to orange for **3** (Figure 5E,F), while the bright green emission was observed for both compounds **2** and **3** under UV light (Figure 5G,H). In contrast, the colors and weak green fluorescence signals of the BODIPY solutions remained unchanged when the foreign species, including, the abovementioned oxidizers, reactive oxygen species (ROS), and oxyanions were added. These results suggested that the triazolopyridine cyclization reactions of the BODIPYs were highly specific toward hypochlorous acid.

**Figure 6:**
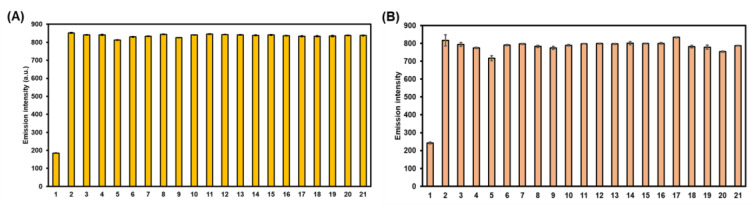
Bar charts demonstrating fluorescence intensities at 512 nm of compounds **2** (**A**) and **3** (**B**) in the presence of HOCl with and without various analytes in MeOH—5 mM PBS (1:1 v/v). [BODIPYs] = 0.2 µM, [HOCl] = 40 µM, [Other analytes] = 40 µM. 1: blank, 2: HOCl, 3: HOCl + H_2_O_2_, 4: HOCl + ^1^O_2_, 5: HOCl + ^·^OH, 6: HOCl + ONOO^-^, 7: HOCl + *t*BuOOH, 8: HOCl + *t*BuO^·^, 9: HOCl + ^·^O_2_^−^, 10: HOCl + Ascorbate, 11: HOCl + L-Cysteine, 12: HOCl + L-Glutathione, 13: HOCl + NO_3_^−^, 14: HOCl + NO_2_^−^, 15: HOCl + SO_4_^2−^, 16: HOCl + SO_3_^2−^, 17: HOCl + S_2_O_5_^2−^, 18: HOCl + S_2_O_3_^2−^, 19: HOCl + HSO_4_^−^, 20: HOCl + CO_3_^2−^, 21: HOCl + HCO_3_^−^. Error bars represent standard deviation (*n* = 3).

To further evaluate the selective performance these probes toward HOCl, the competitive experiments of **2** and **3** in the presence of HOCl combined with and without various potential competing chemical species at the same concentration were carried out in an aqueous methanol solution. As seen in Figure 6, the bar charts clearly indicated that the co-existence of these analytes does not interfere with the HOCl-mediated cyclization reactions of the BODIPY probes, as the fluorescence signals were reasonably enhanced. This incidence served as an additional proof for the selectivity of this HOCl-sensing reaction in the BODIPY backbone without any interfering effect from other oxidizers, ROS, and oxyanions.

In addition, because glutathione (GSH), the major intracellular thiol tripeptide, could be found in high concentrations in most cells [71,72], we decided to examine the effect of high GSH levels toward the HOCl-sensing performance of compounds **2** and **3**. As seen in Figures S15A and S16A, **2** and **3** showed negligible changes in fluorescent intensities in the presence of the high concentrations of GSH from 1 to 10 mM. Moreover, they still underwent a fluorescence turn-on process upon the addition of HOCl in the presence of high levels of GSH (1–10 mM), as displayed in Figures S15B and S16B. These results clearly suggested that the high contents of GSH did not affect the HOCl-detecting ability of these pyridylhydrazone-tethered BODIPY compounds.

### 3.3. Theoretical Calculations

**Figure 7:**
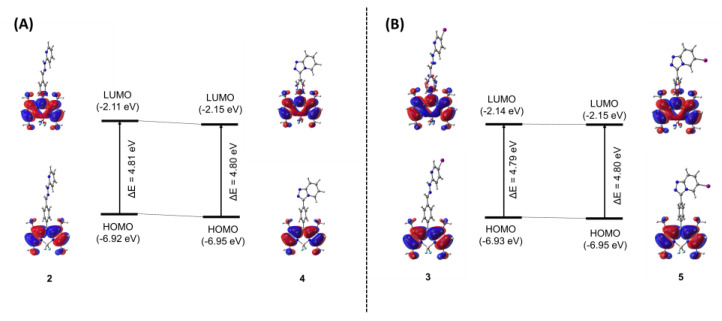
Frontier molecular orbitals (HOMO and LUMO) of compounds **2** and **4** (A), and compounds **3** and **5** (B) and their energy gaps (ΔE in eV) computed by M062X/6-311G* level of theory. The HOMO and LUMO orbitals are plotted with isovalue of 0.02.

The ground state structures of compounds **2**-**5** were optimized using M062X/6-311G* level of theory in the implicit aqueous solution. The frontier molecular orbitals (FMOs) were elucidated to give a detailed picture of the highest occupied molecular orbitals (HOMOs) and the lowest unoccupied molecular orbitals (LUMOs) with the isovalue of 0.02, as well as their energy gaps (HOMO–LUMO energy differences; ΔE) (Figure 7). As depicted in Figure 7A,B, upon the transformation from compounds **2** and **3** to compounds **4** and **5**, both HOMO and LUMO of the cyclization forms (**4** and **5**) were stabilized due to the effect from the extended π-conjugation. In the FMOs, all compounds showed that the electron density distributed solely on the BODIPY backbone in the HOMO, while slightly moved to phenylene linker in the LUMO. The energy gaps (ΔE) of BODIPYs **2**-**5** are quite identical with the values of 4.81, 4.79, 4.80, and 4.80 eV, respectively. These calculations are consistent with the experimental results that all compounds displayed quite similar absorption maxima.

### 3.4. Visualization of HOCl in Living Cells

Before applying compounds **2** and **3** to detect intracellular HOCl, the cytotoxicity profiles of these compounds were evaluated in a murine live macrophage cell line, RAW 264.7. The RAW cells treated with probes **2** and **3** kept their vitality over 85% at doses as high as 50 μM by utilizing the standard MTT assay suggesting great biocompatibility of these compounds. However, when cells are exposed to compounds at a high concentration, such as 100 μM, the cell survival rate falls to around 70% (Figure S13).

**Figure 8:**
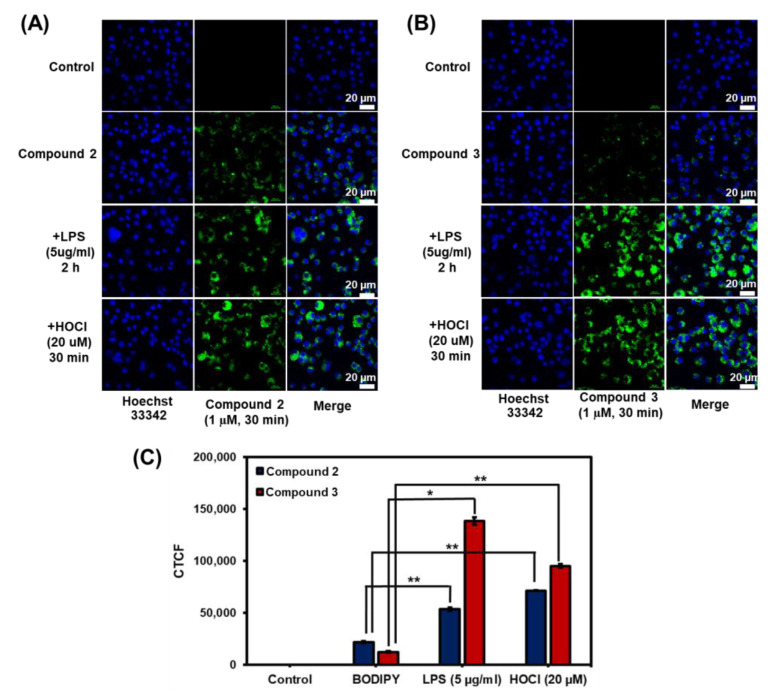
Fluorescence images of compounds **2** and **3** in RAW264.7 cells: (**A**) fluorescence images of compounds **2** and (**B**) compound **3**. For the imaging of endogenous HOCl, RAW 264.7 cells were incubated with 1 μM of the probes for 30 min and then 5 µg/mL of LPS for another 2 h. For the imaging of exogenous HOCl, RAW 264.7 cells were stained with 1 μM of the probes for 30 min and then NaOCl (20 µM) for another 30 min. The images were obtained using a laser scanning confocal microscope (Nikon A1Rsi, 63× oil immersed optics). Scale bar = 20; (**C**) Quantitative corrected total cell fluorescence (CTCF) data, which were quantified using ImageJ and represent the mean ± SD (*n* = 30). Statistical analysis is based on T-test (* *p* < 0.05, ** *p* < 0.01, *** *p* < 0.001).

Next, confocal laser scanning microscopic fluorescence imaging experiments were carried out in murine RAW 264.7 macrophages in the absence and presence of HOCl to validate the cell permeability and capability of compounds **2** and **3** in the detection of HOCl in living cells. In the absence of HOCl, the cells treated with **2** and **3** fluoresced faintly green, as seen in Figure 8A,B. For the detection of endogenous HOCl, the RAW cells were stimulated with lipopolysaccharide (LPS) to produce HOCl prior to the treatment with probes **2** and **3** [73]. Gratifyingly, compound **3** showed green emission signals 11 times higher, while compound **2** demonstrated green fluorescence 2.5 times higher than those in the cells treated with BODIPYs without LPS stimulation (Figure 8C). For exogenous HOCl imaging, compounds **2** and **3** displayed green emission signals after the addition of external HOCl with about 3 times and 8 times higher than those in cells without HOCl treatment, respectively. Notably, the presence of iodine in **3** could lead to higher hydrophobicity of the compound resulting in better membrane permeability [74,75,76]. Additionally, the nuclei of the living cells were stained with Hoechst 33342 to confirm that the green signals of **2** and **3** were created inside the cells but not inside the nucleuses. The Pearson correlation coefficient values, which denoted the degree of overlapping, ranged from 0.13 to 0.22 for all cases, indicating that the probes did not travel to nucleuses (Figure 8 and Figure S17). These findings confirmed the potential of probes **2** and **3** as HOCl detection tools in living cells.

## 4. Conclusions

In summary, the known pyridylhydrazone-linked BODIPY (**2**) and its iodo-substituted analogue (**3**) were successfully proven as highly selective chemosensors for hypochlorous acid (HOCl) through triazolopyridine cyclization of the pyridylhydrazone moiety. The single-crystal X-ray diffraction (SC-XRD), the infrared spectroscopic technique, and the NMR titration experiment confirmed the formation of resulting triazolopyridine-tethered BODIPY derivatives. In addition, the photophysical studies revealed that these two probes could efficiently detect HOCl through a fluorescence enhancement mechanism in MeOH—5 mM PBS (1:1 *v/v*) with the limit of detections of 0.21 µM and 0.77 µM for **2** and **3**, respectively. The DFT calculations disclosed that the change of energy gaps (ΔE) between the open forms and the cyclic forms of the BODIPYs are negligible, which is consistent with the experimental data. Finally, these two compounds displayed a great biocompatibility toward RAW 264.7 cells and could efficiently visualize both endogenous and exogenous HOCl in living cells. It is worth noting that the BODIPY **3**, containing iodo substituent, possesses good cell membrane permeability due to its hydrophobicity.

## Figures and Tables

**Table 1:** Photophysical properties of compounds **2**-**5** in MeOH-5mM PBS (1:1 *v/v*).

Cpd.	Absorption		Emission	
	λ_max_ (nm)	ε (M^−1^cm^−1^)	λ_max_^[a]^ (nm)	Φ_f_^[b]^
**2**	500	8.12 × 10^4^	512	0.08
**3**	500	7.46 × 10^4^	512	0.16
**4**	500	6.78 × 10^4^	512	0.79
**5**	506	5.58 × 10^4^	514	0.77

^[a]^ excitation wavelength = 475 nm. ^[b]^ Φ_f_ were calculated using fluorescein in 0.1M NaOH solution as a standard.

**Table 2:** Comparison of BODIPY-based sensors (compounds **2** and **3**) developed in this work with the recently reported BODIPY-based fluorescence sensors for HOCl/ClO^-^ determination in the literatures.

HOCl Sensor	Working System ^[a]^	Sensing Mechanism	LOD	Ref.
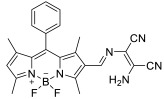	EtOH-10 mM HEPES (1:1 *v/v*)	oxidative cyclization of *γ*-aminoimine	0.12 µM	[65]
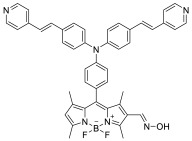	THF—PBS (1:1 *v/v*)	deoximation	0.74 µM	[66]
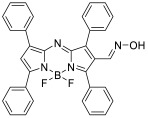	ACN—HEPES (1:1 *v/v*)	deoximation	2.33 µM	[67]
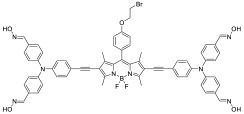	THF–10 mM PBS (1:1 *v/v*)	deoximation	4.9 µM	[68]
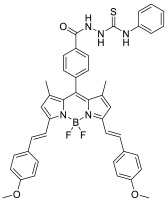	THF—10 mM PBS (1:1 *v/v*)	desulfurization-induced oxadiazole formation	1.15 µM	[69]
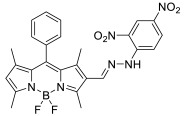	DMF—10 mM PBS (1:1 *v/v*)	Removal of 2,4-dinitrophenyl hydrazine	0.228 µM	[70]
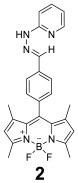	MeOH—5 mM PBS (1:1 *v/v*)	triazolopyridine Formation	0.21 µM	This work
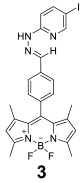	MeOH—5 mM PBS (1:1 *v/v*)	triazolopyridine Formation	0.77 µM	This work

^[a]^ EtOH = Ethanol, THF = Tetrahydrofuran, ACN = Acetonitrile, DMF = Dimethylformamide, HEPES = 4-(2-Hydroxyethyl)-1-piperazineethanesulfonic acid buffer, PBS = Phosphate buffer solution.

## Data Availability

Not applicable.

## Supplementary Materials

The following supporting information can be downloaded at: https://www.mdpi.com/article/10.3390/bios12110923/s1, Figure S1: ^1^H NMR spectrum of **3**; Figure S2: ^13^C NMR spectrum of **3**; Figure S3: ^1^H NMR spectrum of **4**; Figure S4: ^13^C NMR spectrum of **4**; Figure S5: ^1^H NMR spectrum of **5**; Figure S6: ^13^C NMR spectrum of **5**; Figure S7: Mass spectrum of **3**; Figure S8: Mass spectrum of **4**; Figure S9: Mass sprctrum of **5**; Figure S10: Emission intensity of **2** in acetonitrile (ACN), ethanol (EtOH), and methanol (MeOH) before and after adding HOCl; Figure S11: Emission intensity of **2** before and after adding HOCl in MeOH-DI water mixture with various percentages of DI water; Figure S12: FTIR spectra of compound **2** and its HOCl-mediated cyclization product (compound **4**); Figure S13: Cytotoxicity of compounds **2** and **3** were determined using RAW264.7 cells by MTT assay. Statistical analysis is based on T-test (* *p* < 0.05, ** *p* < 0.01, *** *p* < 0.001); Figure S14: Time courses of HOCl-mediated triazolopyridine cyclization reaction of **2** (A) and **3** (B) (0.2 µM) after addition of HOCl (40 mM) in MeOH-5 mM PBS (1:1 *v*/*v*); Figure S15: A) Bar charts demonstrating fluorescence intensities at 512 nm of the sensor (compound **2**) in the presence of GSH (1–10 mM) B) Bar charts demonstrating fluorescence intensities at 512 nm of the sensor (compounds **2**) in the presence of HOCl and GSH (1–10 mM); Figure S16: A) Bar charts demonstrating fluorescence intensities at 512 nm of the sensor (compound **3**) in the presence of GSH (1–10 mM) B) Bar charts demonstrating fluorescence intensities at 512 nm of the sensor (compounds **3**) in the presence of HOCl and GSH (1–10 mM); Figure S17: Pearson correlation coefficient values for colocalization of compound **2** or compound **3** and Hoechst 33342 obtained from ImageJ; Table S1: Crystal data and structure refinement details for compound **4**; Table S2: Comparison of BODIPY-based sensors (compounds 2 and 3) developed in this work with the recently reported rhodol-based fluorescence sensors for HOCl determination via triazolopyridine formation.
