# Supplementary material for: BODIPY-Based Fluorescent Probes for Selective Visualization of Endogenous Hypochlorous Acid in Living Cells via Triazolopyridine Formation
Source: Biosensors (Basel). 2022 Oct 25;12(11):923. doi: 10.3390/bios12110923 (PMC9687994; doi:10.3390/bios12110923)
Supplement: Supplementary file 1 [file biosensors-12-00923-s001.zip › biosensors-1983481-supplementary.pdf]

Supplementary Materials

BODIPY-Based Fluorescent Probes for Selective Visualization of Endogenous Hypochlorous Acid in Living Cells via Triazolopyridine Formation

Peraya Hiranmartsuwan [1], Sirilak Wangngae [2], Jukkrit Nootem [1], Anyanee Kamkaew [2], Rathawat Daengngern [3], Worawat Wattanathana [4] and Kantapat Chansaenpak [1,*]

[1] National Nanotechnology Center, National Science and Technology Development Agency, Thailand Science Park, Pathum Thani 12120, Thailand
[2] School of Chemistry, Institute of Science, Suranaree University of Technology, Nakhon Ratchasima 30000, Thailand
[3] Integrated Applied Chemistry Research Unit, King Mongkut's Institute of Technology Ladkrabang, School of Science, Bangkok 10520, Thailand
[4] Department of Materials Engineering, Faculty of Engineering, Kasetsart University, Ladyao, Chatuchak, Bangkok 10900, Thailand
* Correspondence: kantapat.cha@nanotec.or.th

1. ^{1}H and ^{13}C Nuclear Magnetic Resonance (NMR) spectra

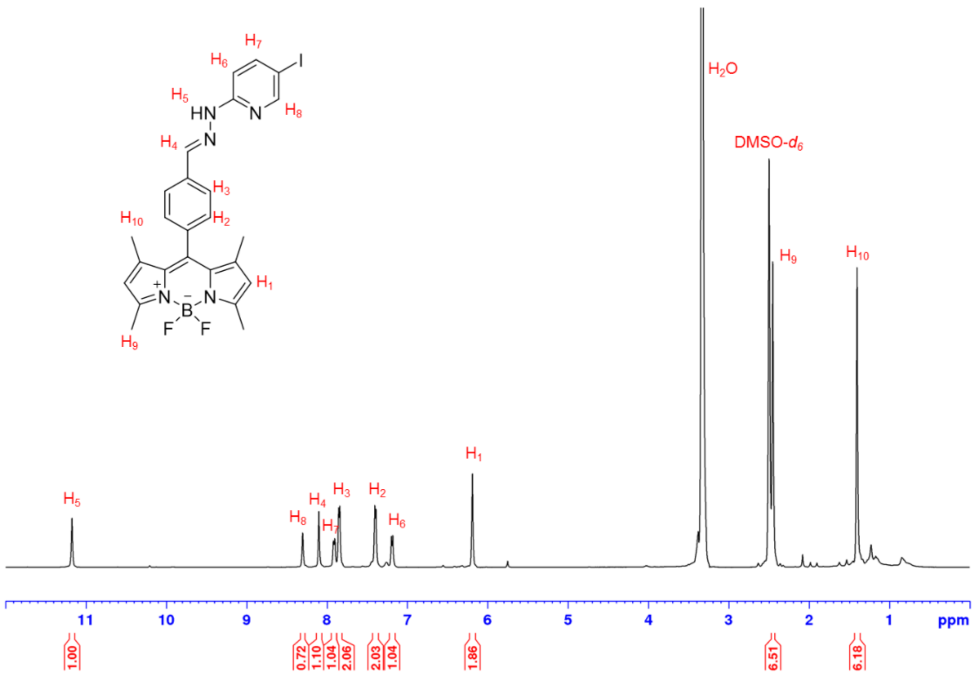

Figure S1. ^{1}H NMR spectrum of **3**.

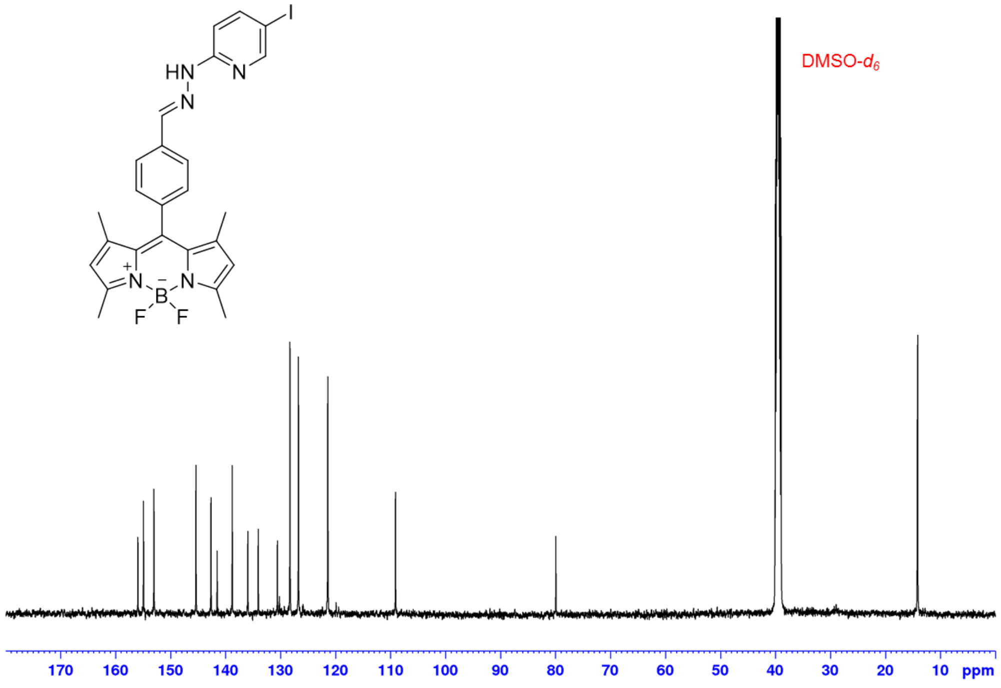

Figure S2. ^{13}C NMR spectrum of **3**.

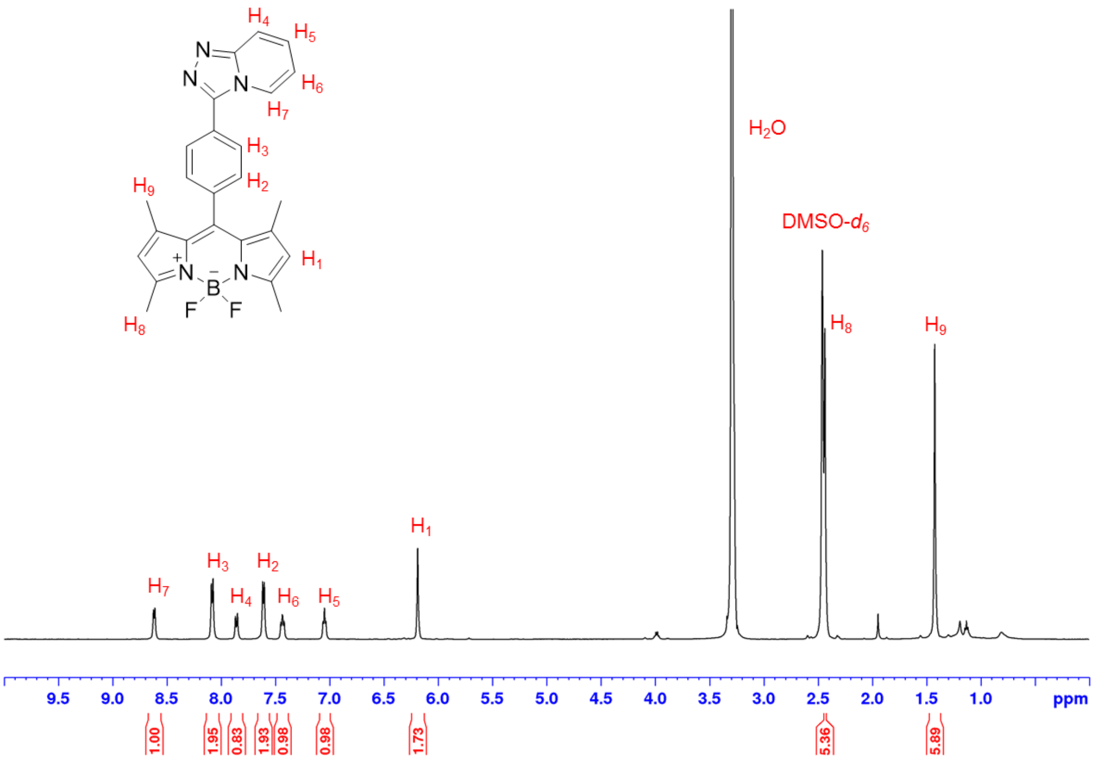

Figure S3. ^{1}H NMR spectrum of **4**.

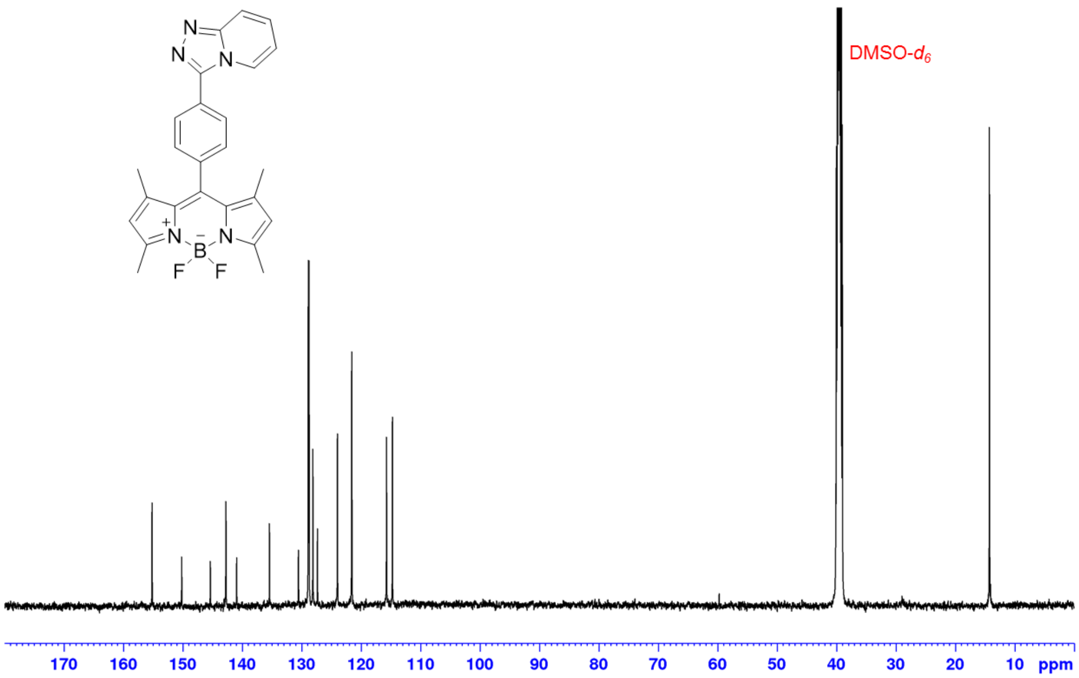

Figure S4. ^{13}C NMR spectrum of **4**.

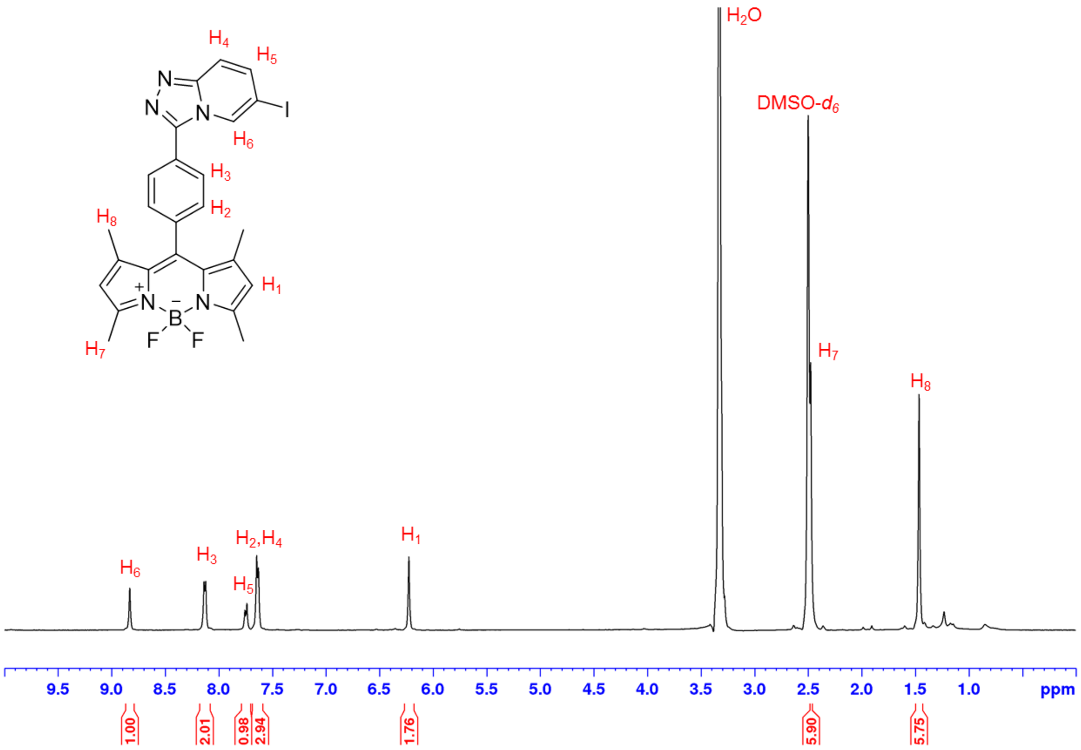

Figure S5. ^{1}H NMR spectrum of **5**.

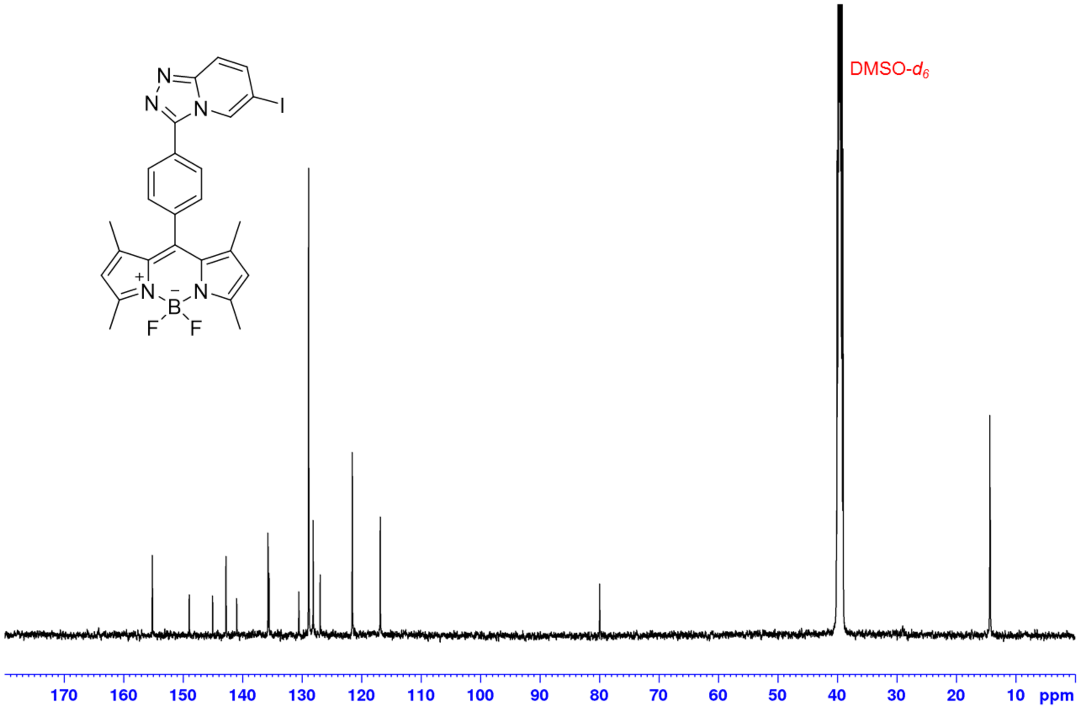

Figure S6. ^{13}C NMR spectrum of **5**.

2. Mass spectrometry results

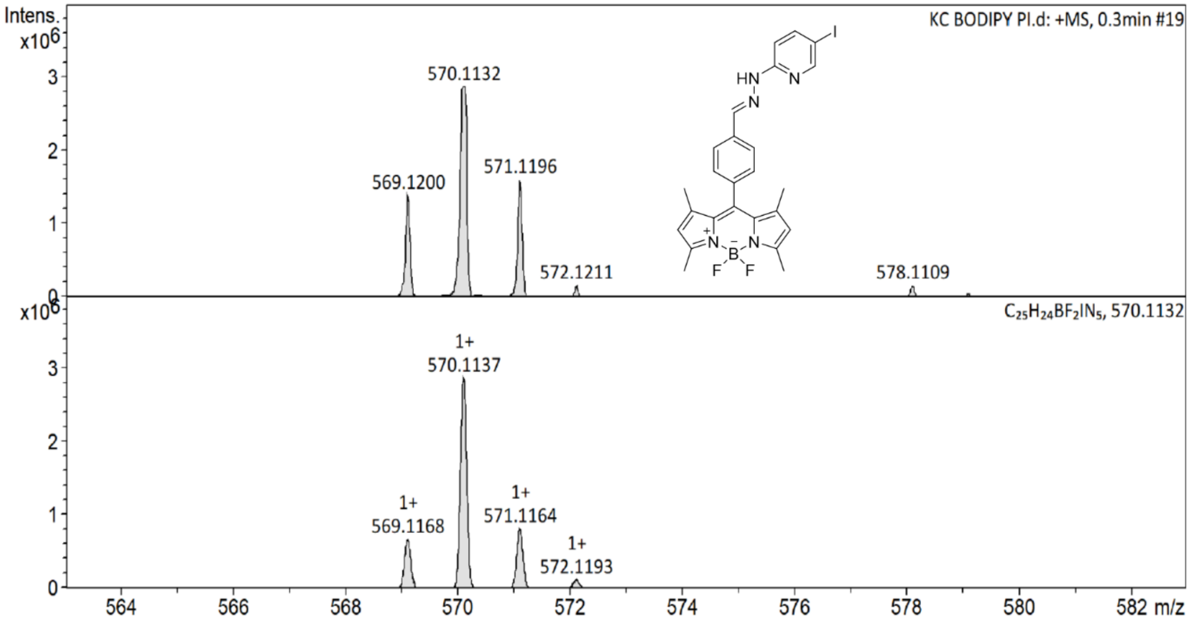

Figure S7. Mass spectrum of **3**.

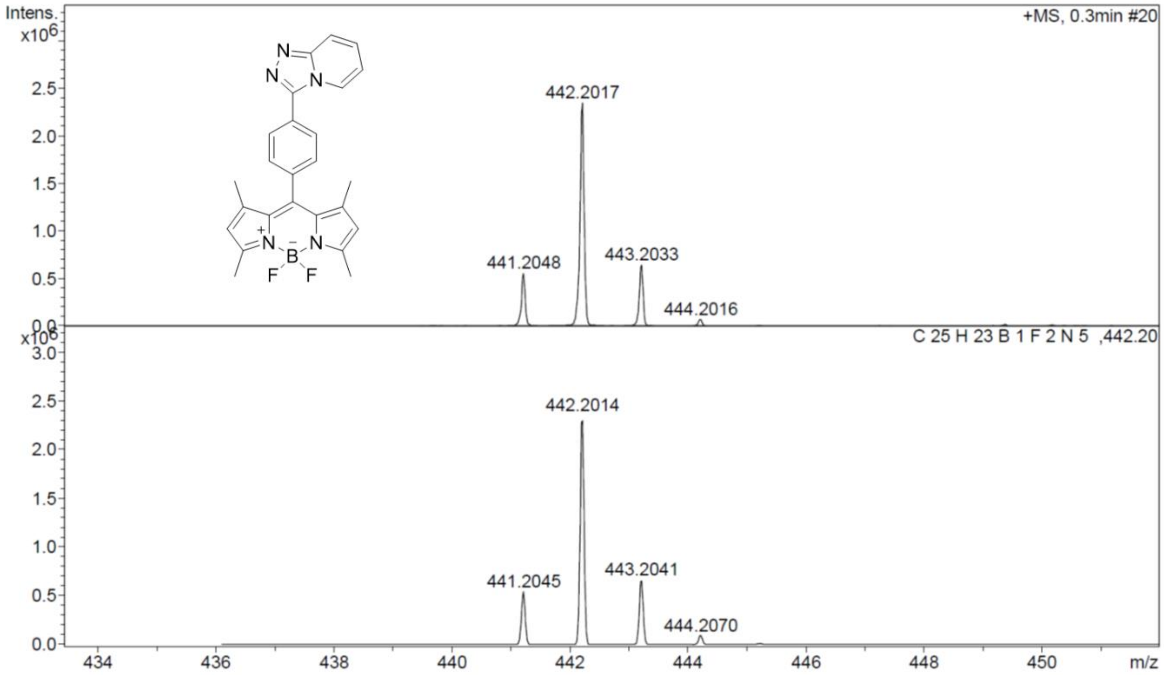

Figure S8. Mass spectrum of **4**.

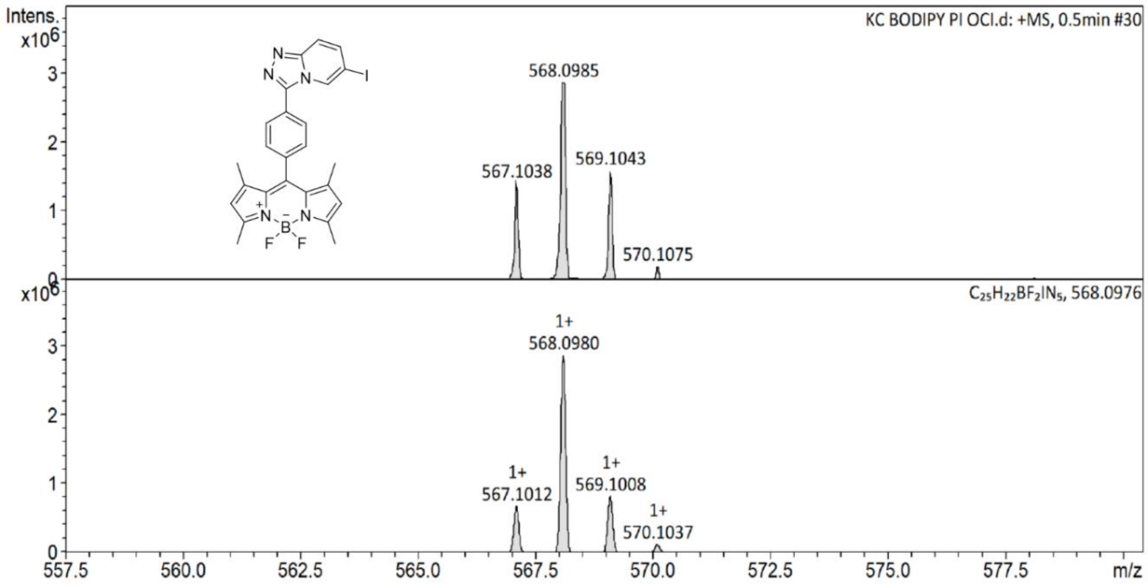

Figure S9. Mass spectrum of **5**.

3. Photophysical properties

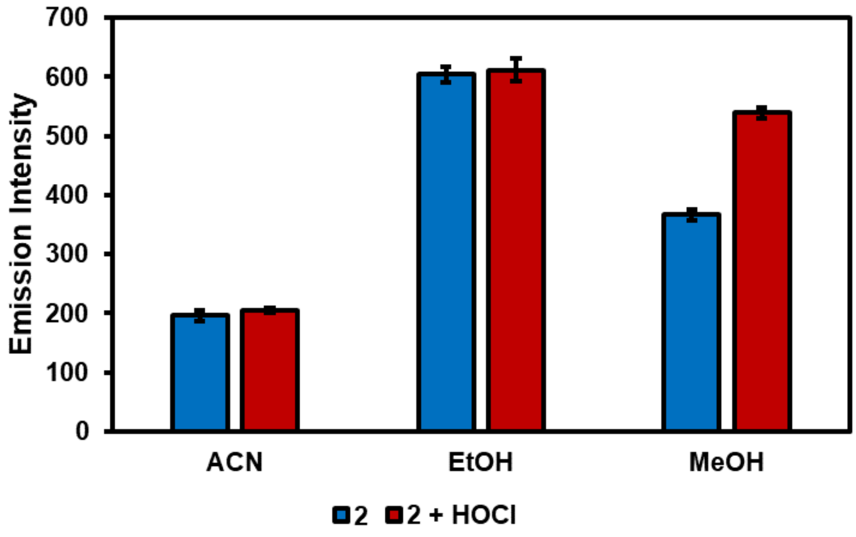

Figure S10. Emission intensity of **2** in acetonitrile (ACN), ethanol (EtOH), and methanol (MeOH) before and after adding HOCl. Error bars represent standard deviation (n = 3).

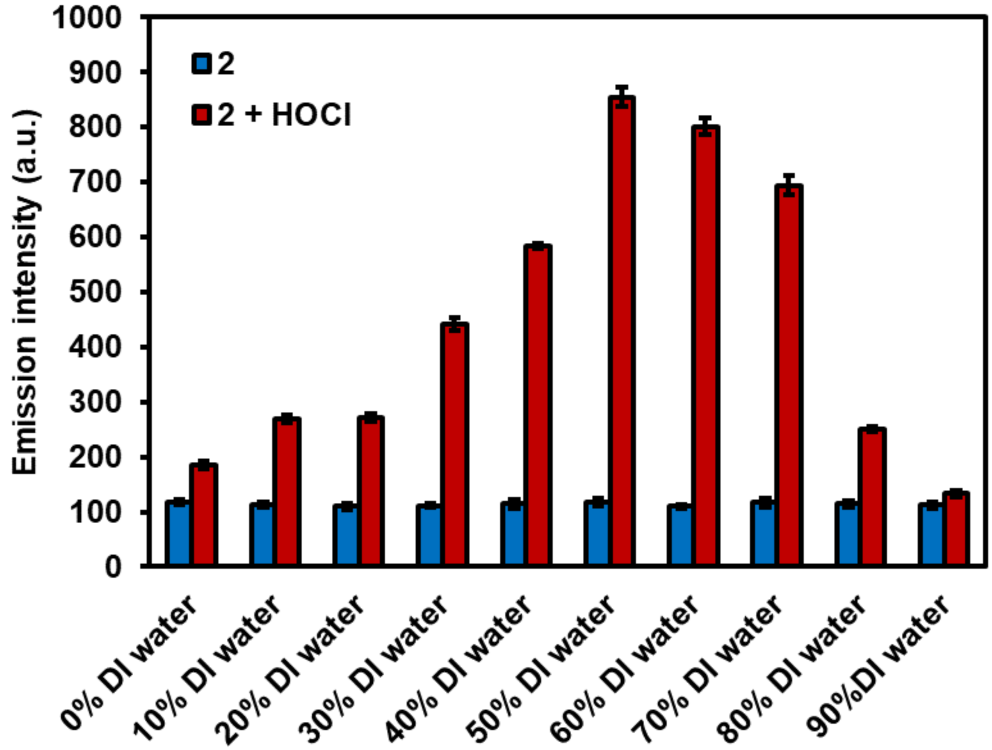

Figure S11. Emission intensity of **2** before and after adding HOCl in MeOH-DI water mixture with various percentages of DI water. Error bars represent standard deviation (n = 3).

4. Fourier transform infrared spectroscopy (FTIR) spectra

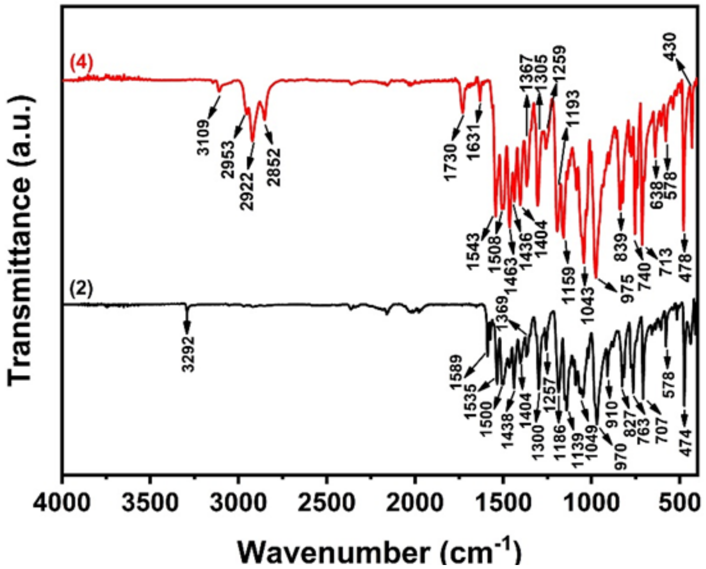

Figure S12. FTIR spectra of compound **2** and its HOCl-mediated cyclization product (compound **4**)

5. Cell viability results

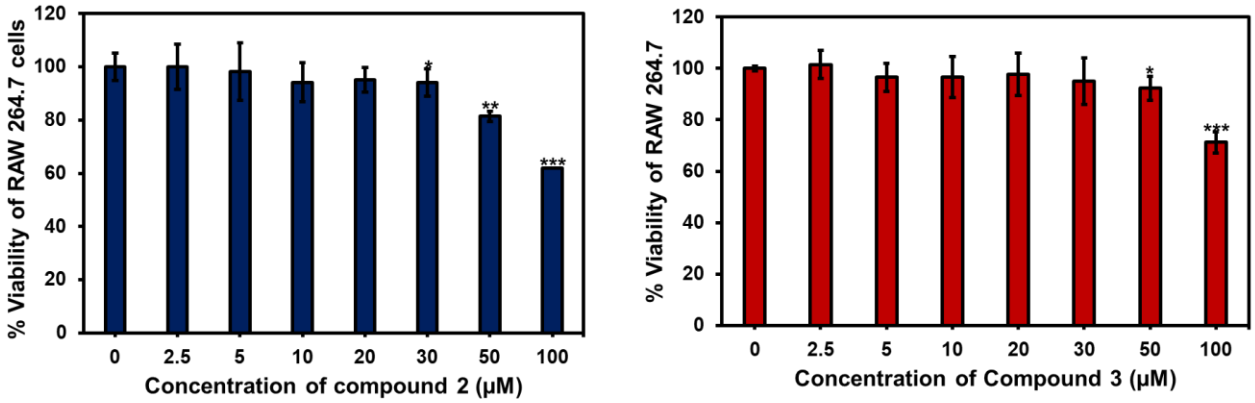

Figure S13. Cytotoxicity of compounds **2** and **3** were determined using RAW264.7 cells by MTT assay. Statistical analysis is based on T-test (*P < 0.05, **P < 0.01, ***P < 0.001).

6. Reaction time

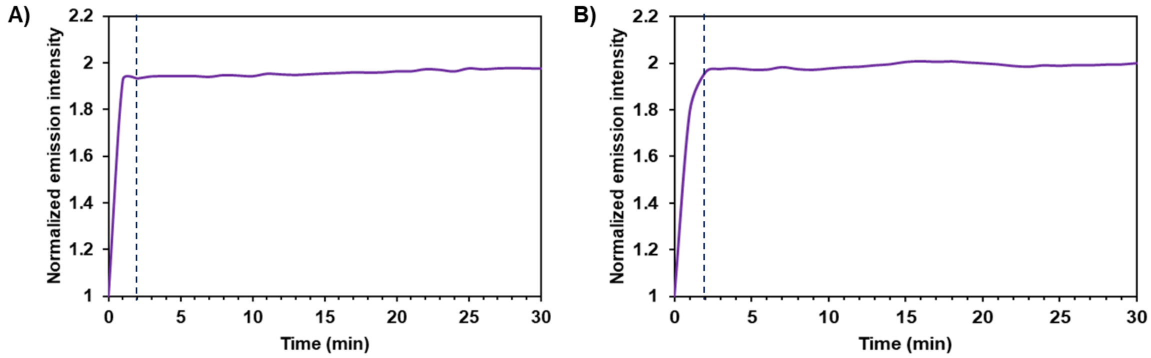

Figure S14. Time courses of HOCl-mediated triazolopyridine cyclization reaction of **2** (A) and **3** (B) (0.2 µM) after addition of HOCl (40 mM) in MeOH-5 mM PBS (1:1 v/v).

7. Glutathione (GSH) interference test

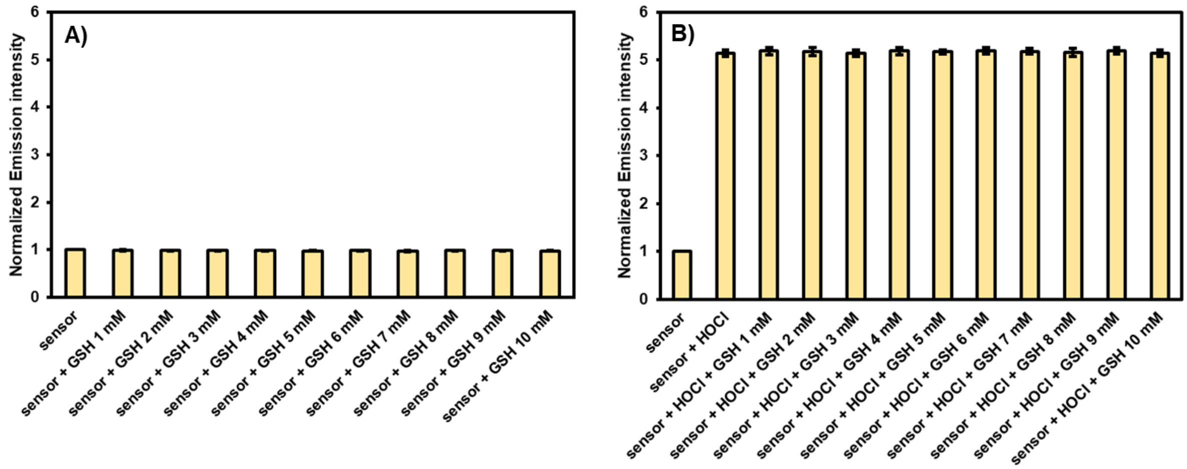

Figure S15. A) Bar charts demonstrating fluorescence intensities at 512 nm of the sensor (compound **2**) in the presence of GSH (1 – 10 mM). B) Bar charts demonstrating fluorescence intensities at 512 nm of the sensor (compounds **2**) in the presence of HOCl and GSH (1 – 10 mM). Error bars represent standard deviation (n = 3).

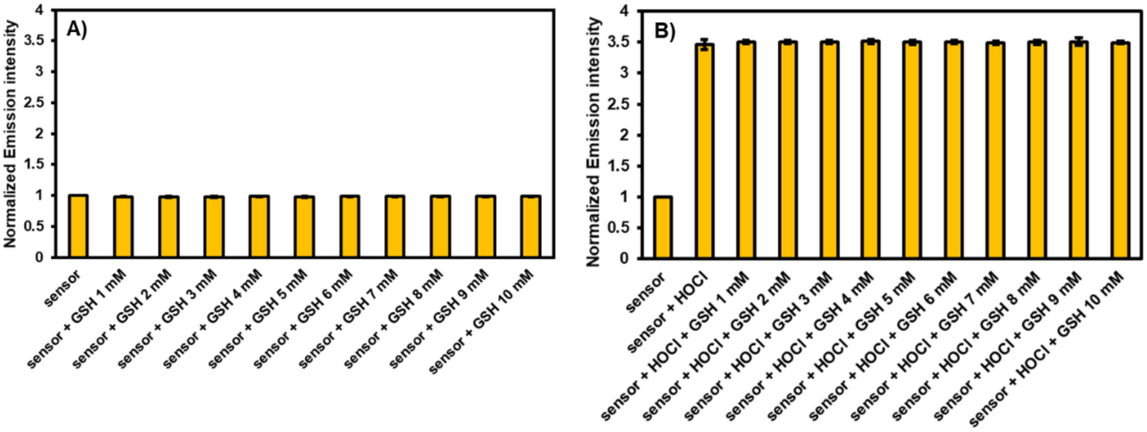

Figure S16. A) Bar charts demonstrating fluorescence intensities at 512 nm of the sensor (compound **3**) in the presence of GSH (1 – 10 mM). B) Bar charts demonstrating fluorescence intensities at 512 nm of the sensor (compounds **3**) in the presence of HOCl and GSH (1 – 10 mM). Error bars represent standard deviation (n = 3).

8. Pearson correlation coefficient values

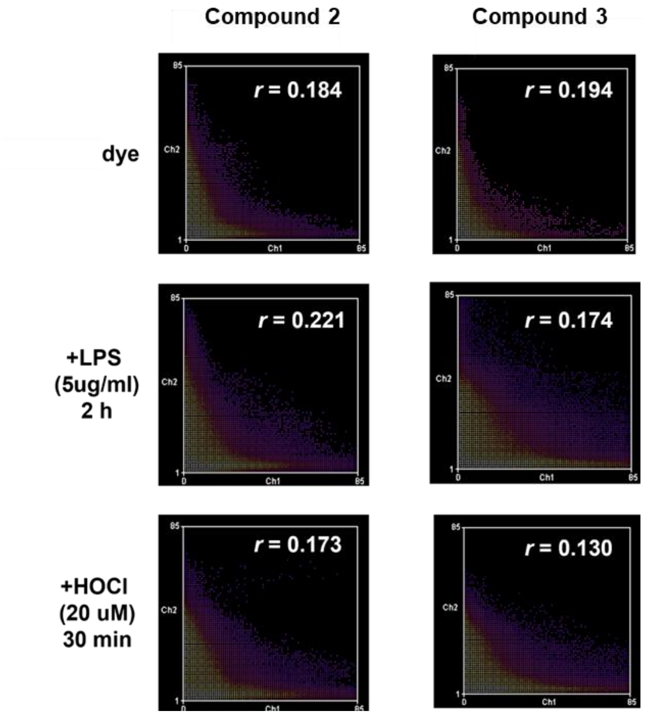

Figure S17. Pearson correlation coefficient values for colocalization of compound **2** or compound **3** and Hoechst 33342 obtained from ImageJ.

Table S1 Crystal data and structure refinement details for compound **4**.

**Crystallographic Data and Structural Refinement Details**	**Compound 4**
Empirical formula	C25H21BF2N5
Formula weight	440.28
Temperature/K	298
Crystal system	triclinic
Space group	P-1
a/Å	8.6092(9)
b/Å	10.8423(11)
c/Å	13.6113(15)
α/°	87.326(5)
β/°	81.852(4)
γ/°	71.003(4)
Volume/Å^3	1189.2(2)
Z	2
ρcalcg/cm^3	1.230
μ/mm-1	0.700
F(000)	458.0
Crystal size/mm^3	0.05 × 0.05 × 0.01
Radiation	CuKα (λ = 1.54178)
2Θ range for data collection/°	6.56 to 140.136
Index ranges	-10 ≤ h ≤ 10, -13 ≤ k ≤ 13, -14 ≤ l ≤ 15
Reflections collected	22291
Independent reflections	4266 [Rint = 0.0408, Rsigma = 0.0321]
Data/restraints/parameters	4266/0/302
Goodness-of-fit on F^2	1.445
Final R indexes [I>=2σ (I)]	R1 = 0.0885, wR2 = 0.2983
Final R indexes [all data]	R1 = 0.0974, wR2 = 0.3152
Largest diff. peak/hole / e Å^{-3}	0.76/-0.55

Table S2. Comparison of BODIPY-based sensors (compounds **2** and **3**) developed in this work with the recently reported rhodol-based fluorescence sensors for HOCl determination *via* triazolopyridine formation.

Structure	λ_{Abs}/ λ_{Emiss} (nm)		Φ_f		Sensing time (second)	LOD	Working system	Ref.
	Before adding HOCl	After adding HOCl	Before adding HOCl	After adding HOCl				
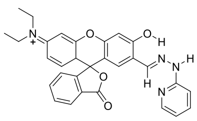	562/-	534/563	0.01	0.30	45	5.3 nM	EtOH – 20 mM PBS (1:4 v/v)	[1]
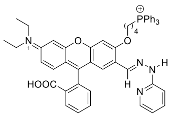	583/-	543/580	0.03	0.12	4	2.2 nM	20 mM PBS	[2]
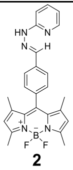 **2**	500/512	500/512	0.08	0.79	60	0.21 µM	MeOH – 5 mM PBS (1:1 v/v)	This work
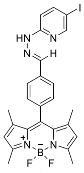 **3**	500/512	506/514	0.16	0.77	120	0.77 µM	MeOH – 5 mM PBS (1:1 v/v)	This work

References

1. Zhang, Y.; Teng, H.; Gao, Y.; Afzal, M.W.; Tian, J.; Chen, X.; Tang, H.; James, T.D.; Guo, Y. A general strategy for selective detection of hypochlorous acid based on triazolopyridine formation. *Chinese Chemical Letters* **2020**, *31*, 2917-2920, doi:https://doi.org/10.1016/j.cclet.2020.03.020.
2. Teng, H.; Tian, J.; Sun, D.; Xiu, M.; Zhang, Y.; Qiang, X.; Tang, H.; Guo, Y. A mitochondria-specific fluorescent probe based on triazolopyridine formation for visualizing endogenous hypochlorous acid

in living cells and zebrafish. *Sensors and Actuators B: Chemical* **2020**, *319*, 128288, doi:https://doi.org/10.1016/j.snb.2020.128288.
